# Recurrent Carotid artery blow out in a head & neck patient

**DOI:** 10.1016/j.ijscr.2022.107089

**Published:** 2022-04-18

**Authors:** Cara Íosa Harrington, Nicholas O'Keeffe, Paul Lennon

**Affiliations:** Department of Otolaryngology/Head & Neck Surgery, St James' Hospital, Dublin 8, Ireland

**Keywords:** SCC, Squamous Cell Carcinoma, CBS, Carotid Blowout Syndrome, CT, Commuted Tomography, HBOT, Hyperbaric Oxygen Therapy, Case report, Carotid artery blowout, Head and neck cancer, Surgical emergency

## Abstract

**Introduction and importance:**

Carotid artery blowout syndrome is a rare complication of head and neck cancer treatment. It defines a rupture of the carotid artery wall through vessel wall necrosis. This is typically precipitated by radiotherapy, direct tumour invasion, or a combination of these factors. We describe a rare case of three consecutive carotid artery blowouts in a head and neck cancer patient.

**Case presentation:**

A 58-year-old man with a history of T3NO hypopharyngeal squamous cell carcinoma (SCC) treated with chemotherapy and radiation presented with a four-month history of progressive dysphagia and right sided neck pain. Flexible nasendoscopy revealed laryngeal oedema and slough. A panendoscopy and biopsy showed no evidence of tumour recurrence. The patient was discharged and represented with worsening dyspnoea. He subsequently experienced a large volume hemorrhage necessitating ligation of his right external carotid artery. He underwent pharyngolaryngectomy indicated due to the extent of laryngeal radiation necrosis. Thereafter he suffered two additional acute carotid bleeds from his right common carotid necessitating ligation in theatre.

**Clinical discussion and conclusion:**

This case report illustrates the key issues to be considered in patients with locally advanced hypopharyngeal squamous cell carcinoma and subsequent management of acute carotid blowout syndrome, which without prompt management, can be fatal.

## Highlights

• Carotid artery rupture, or blowout, has a high morbidity and mortality.
• Carotid blowout is a rare consequence of head and neck cancer treatment.
• Radiotherapy is the main predisposing risk factor.
• Acute carotid blowout requires prompt resuscitation and bleeding source control.
• Definitive treatment includes endovascular occlusion, stenting or surgical ligation.

## 1 Introduction

Carotid artery blow out syndrome (CBS) is a rare but often fatal complication of head and neck cancer treatment. It is the rupture of the carotid artery secondary to vessel wall necrosis often precipitated by primary irradiation, or adjuvant radiation in head and neck cancer patients. In patients who have undergone head and neck irradiation; wound breakdown, pharyngocutaneous fistula and infection further perpetuate this risk [1]. Tumour recurrence and progression additionally predispose patients to CBS. Acute CBS carries a high morbidity and mortality necessitating prompt resuscitation [2], [3]. Herein we present the only reported case of three consecutive carotid artery blow outs in a head and neck cancer patient. This case report has been reported in line with the SCARE criteria [4].

## 2 Case presentation

**Fig. 1:**
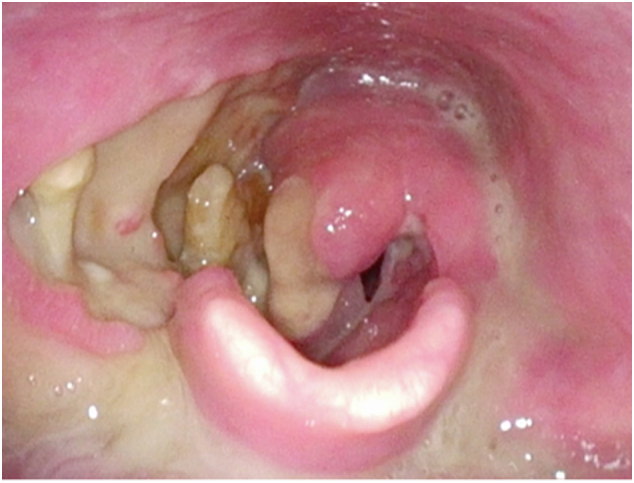
Flexible nasendoscopy showing evidence of radionecrosis.

A 58-year-old male with a history of T3NO hypopharyngeal squamous cell carcinoma (SCC), on a background of smoking and alcohol use, treated with chemotherapy and radiation nine months prior re-presented to a tertiary referral centre for head and neck cancer clinic with a four-month history of progressive dysphagia and right sided neck pain. Examination with flexible nasendoscopy revealed largyngeal oedema and extensive sloughing, exposed laryngeal cartilage, but no definite evidence of a recurrent tumour (Fig. 1).

A panendoscopy was performed, and biopsy of the right lateral pharynx, which showed no evidence of tumour recurrence. The patient was discharged and subsequently represented with worsening symptoms of dysphagia, dyspnoea and pain a month later. Repeat panendoscopy demonstrated further cartilage exposure, but again no evidence of recurrent malignancy, despite progression of the patient's clinical symptoms. The patient was admitted to hospital for analgesia and observation. Five days later he experienced a large volume hemorrhage necessitating neck exploration and ligation of his right external carotid artery and tracheostomy carried out by the head and neck surgical consultant. The patient subsequently underwent a pharyngolaryngectomy with gastric pull up and primary anastomosis of conduit to pharynx, indicated due to the extent of radiation induced laryngeal necrosis. His post-operative course was complicated by an anastamotic leak and a salivary pharyngocutaneous fistula. This was initially managed conservatively, however three weeks later the patient suffered a further massive acute hemorrhage requiring resuscitation, and emergent return to the operating theatre. On this occasion, a defect in his right common carotid artery which was repaired using a biopatch with the assistance of vascular surgeons. The pharyngocutaneous fistula was closed by the head and neck surgeons with a rolled myocutaneous pectoralis major flap, with a supraclavicular flap required to close the skin defect. Eleven days later the patient experienced a third massive bleed in the absence of further salivary fistula. Following resuscitation, and in consultation with a vascular surgeon, on this occasion the right common carotid artery was ligated. No further bleeding occurred following this intervention. Subsequent imaging revealed an ischaemic stroke with right parieto-temporal watershed regions. Clinically, this manifested with left sided neglect with no major motor deficit. After a prolonged rehabilitation, the patient was discharged, on normal diet and has returned to most normal daily activities, including employment.

## 3 Discussion

The patient in this report was initially treated with primary chemoradiotherapy, however, radiation-induced soft tissue necrosis influenced his subsequent clinical course. Multiple re-biopsies did not identify disease recurrence.

Approximately 75% of patients with head and neck cancer may undergo curative or palliative radiotherapy [5]. Notwithstanding improvements in radiation planning and execution, a large proportion develop radiotherapy- related toxicity amplified further by systemic chemotherapy [6].

Radiation necrosis is a late and typically irreversible complication [7]. Necrosis may be evaluated with Commuted Tomography (CT), however this cannot distinguish radiation necrosis from tumour recurrence. Direct visualisation of laryngeal sloughing with tissue biopsy may enhance diagnostic specificity [8], [9], [10].

Severe necrosis can manifest with respiratory distress, dysphagia, and odynophagia. Surgical intervention with tracheostomy and total laryngectomy is typically indicated. Hyperbaric oxygen therapy (HBOT) has shown comparable results to laryngectomy in the treatment of laryngeal radionecrosis, with improved function and pain scores [11], [12].

Radiotherapy predisposes head and neck cancer patients to an eight-fold risk of CBS development, representing the most significant predisposing factor [13], [14]. Likewise, eighty to 90% of patients who develop CBS have a history of radiotherapy to the head and neck [1]. Long-term tracheostomies, infection, fistula, poor wound healing and previous neck dissection further predispose patients to CBS [15].

Acute CBS represents an acute threat to a patient's life. Management depends on the nature and acuity of bleeding. In the present case, the patient experienced three separate acute carotid blowouts necessitating advanced cardiac life support. Survival and achieving haemostasis in acute CBS depend on initial effective resuscitation and bleeding source control [15], [16]. Once patients are stabilised, angiography is favoured for its diagnostic and therapeutic propensities [17].

Surgical ligature and endovascular occlusion or repair with covered stents can control bleeding [1]. Stents may reduce neurological morbidity, but are associated with higher rates of infection and rebleeding [18]. In centres lacking immediate interventional facilities, and in patients with external carotid artery rupture, ligation may be performed.

Ligation represents an anatomical challenge owed to by previous anatomical site irradiation and/or infected tissues, but may prevent recurrent CBS [19], [20]. However, one must acknowledge a 15–100% rate of mortality with permanent neurological morbidity in 10–20% of patients [21].

A palliative approach may be most appropriate in those with metastatic malignancies, in those without quick access to appropriate medical support, in centres without appropriate surgical or anaesthetic support, or in patients who choose not to seek active resuscitation [22].

In patients who survive CBS, three year survival decreases from 100% to 12% in the presence of active carcinoma [23]. Thus, management with a curative intent was desired in this case as multiple biopsies failed to demonstrate histological evidence of cancer recurrence.

## 4 Conclusion

This is the first published report of three consecutive carotid artery blow outs in a head and neck cancer patient. Our experience highlights the clinical decision making process when managing soft tissue radiation induced necrosis of the hypopharynx and the subsequent management of carotid artery blow outs.

## Provenance and peer review

Not commissioned, externally peer-reviewed.

## Sources of funding

N/A.

## Ethical approval

N/A.

## Consent

Written informed consent was obtained from the patient for the publication of this case.

## Research registration number

N/A.

## Guarantor

Mr. Paul Lennon at St James' Hospital, Dublin 8, Ireland.

## CRediT authorship contribution statement

Harrington C: Study Concept, data collection and writing paper.

O'Keeffe N: Study Concept, data collection, writing of paper and advised report.

Lennon P: Writing paper and advised report.

## Declaration of competing interest

No potential conflict of interest relating to this article have been reported.
